# Vaccination in pregnancy: The role of the midwife

**DOI:** 10.3389/fgwh.2022.929173

**Published:** 2022-10-24

**Authors:** Caroline S. E. Homer, Nasrin Javid, Kellie Wilton, Zoe Bradfield

**Affiliations:** ^1^ Maternal, Child and Adolescent Health Program, Burnet Institute, Melbourne, VIC, Australia; ^2^ Faculty of Health, University of Technology Sydney, Sydney, NSW, Australia; ^3^ Nursing and Midwifery Research Alliance, South West Sydney Local Health District, Liverpool, NSW, Australia; ^4^ Australian College of Midwives, Canberra, ACT, Australia; ^5^ Faculty of Health Sciences, King Edward Memorial Hospital and Curtin University, Perth, WA, Australia

**Keywords:** maternal health, vaccination, midwife as a health service provider, pregnancy, influenza, pertussis, tetanus, COVID-19

## Abstract

Midwives are the front-line workers providing maternity care for women in many countries. The role of the midwife includes providing information about, and recommendations for, maternal vaccination in pregnancy and for the baby in the postnatal period. Vaccinations recommended in pregnancy include those to prevent influenza, pertussis, tetanus and now COVID-19. Vaccinations for the newborn baby include hepatitis B. Healthcare professionals play an important role in influencing decision-making around vaccination and midwives are key in supporting vaccination uptake. Midwives are strong influencer in women's decisions around vaccination for themselves and their babies. The COVID-19 vaccination programs have shone a light on vaccination in pregnancy especially as SARS-COV-2 infection has significant adverse effects in pregnancy. COVID-19 vaccination has been shown to be safe and effective in pregnancy. Despite this, there is vaccine hesitancy from pregnant women in many countries. Midwives play a unique role in the provision of care to women and families but they need specific support and information regarding vaccination in pregnancy. Targeted education, supportive mentoring and supervision and opportunities to lead innovative ways of ensuring vaccine access is logistically easy and possible are all needed. This Commentary outlines the key vaccinations recommended in pregnancy including COVID-19 vaccination and highlights some strategies to scale-up vaccination programs in pregnancy with a particular focus on the role of midwives.

## Introduction

In most countries, midwives play a key role in the provision of maternal and newborn health care (1). The international definition of the midwife includes the provision of “support, care and advice during pregnancy, labour and the postpartum period, to conduct births on the midwife's own responsibility and to provide care for the newborn and the infant” (2). The role of the midwife also includes preventative measures as well as health counselling and education, not only for the woman, but also within the family and the community (2). It is these preventative and health counselling roles that highlight the important public health work that midwives lead in promoting vaccination in pregnancy and for the newborn.

Vaccination in pregnancy has always been important, however, the COVID-19 pandemic has shone a brighter light on this issue. Depending on the context, vaccination for tetanus (3), influenza (4, 5) and pertussis (3, 4) is recommended during pregnancy (6) and vaccination for hepatitis B is promoted for the newborn (7). Prior to the COVID-19 pandemic, women had reported concerns about vaccination in pregnancy and many did not feel that they received enough information during pregnancy (8, 9).

Midwives are key to the uptake of vaccination in pregnancy. A systematic review of 45 studies related to uptake of influenza vaccination during pregnancy showed that positive vaccination recommendations from health care providers as well as direct access to the vaccine would improve vaccination acceptance (10). A more recent mini-review showed that the main facilitator for uptake of influenza and pertussis vaccinations among pregnant women was receiving a recommendation from their health care providers (11).

Health worker recommendation is a significant factor in women choosing to have vaccinations in pregnancy (12). For example, in the United Kingdom, vaccination rate for pertussis and influenza was significantly higher among pregnant women who attended a midwife-led immunisation clinic and received vaccination information and recommendations from midwives (13). In Australia, the antenatal care providers' advice (often a midwife) to have the recommended vaccinations in pregnancy was the single most important factor associated with vaccination (8, 14–16). In another Australian study, women who had received a recommendation to have the influenza vaccine from a health care worker were 20 times more likely to get vaccinated (9). Despite this, at a global level, most health care providers still do not recommend some vaccines, like the influenza vaccination, to pregnant women (10) and midwives seem to be more likely to express safety concerns than other health care providers, and less likely to recommend vaccination for infections such as influenza and pertussis (11). These studies highlight the importance of providing health care providers, especially midwives, with the information and agency to make clear recommendations to women and families.

This first aim of this Commentary is to outline the key vaccinations recommended in pregnancy including COVID-19 vaccination. The second aim is to highlight the strategies to scale-up vaccination programs in pregnancy with a particular focus on the role of midwives.

## Vaccines recommended for pregnant women

This section briefly outlines the key vaccinations that are offered to women during pregnancy.

### Influenza

Pregnant women are at higher risk of having severe influenza during pregnancy compared to non-pregnant women, due to the physiological and immunological changes that occur during pregnancy. A literature review demonstrated that the risk of cardiopulmonary complications and admission to hospital is higher, particularly in the second or third trimester (17). During the H1N1 pandemic in 2009 in Australia, pregnant women more than 20 weeks gestation were 13 times more likely to be admitted to an intensive care unit, particularly those who had pre-existing risk factors such as asthma and obesity (18) than non-pregnant women. The study also showed perinatal mortality of 12% (7/60), including four stillbirths and 3 neonatal deaths. Around one-third of babies (37%) were born preterm, and half (57%) were admitted to neonatal intensive care unit or special care nursery (18). These rates were all higher than the background population risks.

Vaccination for influenza has been shown to be safe in pregnancy and the most effective strategy to protect women and reduce the burden of disease on women and their babies less than 6 months old (17, 19). A systematic review of 679,992 pregnant women demonstrated a significant reduction in the rate of preterm birth (<37 weeks) and very preterm birth (<32 weeks) among women who had the influenza vaccine during pregnancy compared to those who were unvaccinated (20). The review found no association between the influenza vaccine and adverse neonatal outcomes. In addition, the rate of anti-influenza antibody was significantly higher among mothers who had a vaccine.

Influenza vaccination at any stage of pregnancy is recommended in many high-income countries. These include Australia (19), Canada (21), New Zealand (22), United States (23), United Kingdom (24), and some countries of Europe (25) by many obstetric colleges and organisations including the Royal Australian and New Zealand College of Obstetricians and Gynaecologists (5) and the American College of Obstetrics and Gynecology (3) and the World Health Organization (26).

### Tetanus

Tetanus is a bacterial infection which can be prevented through vaccination (27). Global elimination of tetanus has been one of the priorities of the World Health Organization (WHO) since 1989 (28). Women in the early days of giving birth and during early postnatal period, as well as neonates are at higher risk of tetanus infection and associated mortality and morbidity (27, 28).

Tetanus toxoid vaccination is recommended for all pregnant women to prevent neonatal mortality from tetanus (29). If the information about the tetanus vaccinations prior to pregnancy is not known, two doses of tetanus-diphtheria is recommended during pregnancy. The interval should be at least 2 weeks with the second dose at least 2 weeks before birth (28).

### Pertussis

The WHO has identified that “vaccination of pregnant women is effective in preventing disease in infants who are too young to be vaccinated” but has not gone so far as to recommend vaccination for all pregnant women (30). In Australia, a single dose of combined diphtheria, tetanus, accellular pertussis (dTpa) vaccine is recommended during pregnancy between second and third trimester, preferably between 20 and 32 weeks gestation (19). In the United Kingdom, a single dose of combined pertussis, diphtheria, tetanus, and polio (called Boostrix) is recommended between second and third trimester, preferably between 16 and 32 weeks gestation (31). The Center for Disease Control in the United States recommences vaccination for pertussis between 27 and 36 weeks of pregnancy, preferably closer to 27 weeks (32).

### COVID-19

COVID-19 vaccination is recommended for women who are pregnant, breastfeeding, trying to get pregnant now, or who might become pregnant in the future. This is because COVID-19 is associated with increased risk of stillbirth, preterm birth, a higher risk of admission to the intensive care unit or needing invasive ventilation, a higher risk of maternal death (33, 34).

COVID-19 vaccination in those who are pregnant or breastfeeding is immunogenic, does not cause significant vaccine-related adverse events or obstetrical and neonatal outcomes, and is effective in preventing COVID-19 disease (35–38).

## Key strategies to scale-up vaccination programs in pregnancy

A number of interventions have been shown to support the scale up of vaccination in pregnancy. A systematic review of 26 articles designed to examine influenza vaccination uptake in pregnancy examined the effective factors from the perspective of three psychological propositions: thoughts and feelings, social processes and changing behaviour directly (39). “Thoughts and feelings” recognises that the decision to get vaccinated is an individual decision. Programs and health care providers must address how people view the disease itself (risk appraisal), what they feel about the effectiveness of the vaccine and their safety concerns (confidence) and the motivation to receive the vaccine (40). “Social processes” recognise that there are wider social issues at play including the relationship between the women and her care provider, her social networks and the social norms about vaccination (40). “Changing behaviour directly” includes having systems and processes that make it easy to access vaccination and harder to forget. Interventions, such as reminders (before the vaccination is due) or recall (when the vaccination is overdue) and provider prompts in electronic medical records serve as a nudge, especially to the health care provider (41). Standing orders which require an “opt-out” rather than an “opt in” approach takes the onus for recommending the vaccine out of the health care providers' hands and has been shown to be effective (42). “Changing behaviour directly” also includes ready access to the vaccination so that again, the uptake is more easy than difficult.

Interventions such as provider prompts and standing orders, known as nudge-based interventions, have been widely used in vaccination programs (39). These still require a dialogue between patient and providers, especially midwives in the case of vaccination during pregnancy, to be effective. One Australian study aimed to address this issue by developing an intervention known as P3-MumBubVax to optimise midwives' vaccine discussions with expectant parents and improve uptake of maternal and childhood vaccines. The study used qualitative approaches from midwives, as well as a review of theoretical models and vaccine communication tools to develop the intervention (43). P3-MumBubVax includes components at three levels: (1) Practice (“vaccine champions”; stickers to prompt and record vaccine discussions/delivery); (2) Provider (website with vaccine communication training; learning exercise; fact sheets; links to child vaccination resources); (3) Parent (SMS reminders; website; fact sheets) (44). This multi-pronged approach is feasible and acceptable and further work is now examining effectiveness (44).

A recent literature review of 157 publications and 117 documents demonstrated a five-pillar framework to describe key components of increasing vaccination uptake in pregnancy. These five pillars are (1) Health authority accountability and strengths of the pregnancy vaccination programme; (2) Facilitated access to vaccination; (3) Healthcare professional accountability and engagement; (4) Awareness of the burden and severity of diseases; (5) Belief in pregnancy vaccination benefits (45). A study in the UK showed that a midwifery-led immunisation clinic increased vaccination rate for influenza and pertussis by addressing these issues. In this study the vaccination rate was 91% for pertussis and 79% for influenza (13). Midwives' recommendation, trust in the information received and ease of access contributed to the increased vaccine uptake by women participating in this study.

Midwifery care during the childbearing period increases health-seeking behaviours and is a key focus of public health policy. Cocoon immunisation is a strategy that supports the vaccinated pregnant woman to encourage vaccine uptake in family members in order to protect the newborn baby at birth. This public health intervention has demonstrated effectiveness in increasing vaccination uptake in pregnant women utilising the powerful influence of the woman's role as a leader in her family. An important component of successful cocoon immunisation is funding to support free, point-of-service access to broader family immunisation (46). Cocoon immunisation, supported by midwives enables woman-centred and mother-led vaccination uptake and is an important strategy with demonstrated benefits to newborns, families, communities and broader society (47).

## The role of the midwife

While health promotion is part of the role of the midwife, specific training to support vaccination in the course of routine education provided to women in pregnancy is often limited. One Australian study showed that midwives had received minimal or no training on vaccine communication and their practice focused primarily on vaccine information provision rather than recommendation/persuasion, although some midwives shared personal views and actively encouraged vaccination (48). Another Australian study using an online survey also showed that the majority of midwives supported maternal (influenza 83%, pertussis 90.5%) and childhood immunisation (85.8%) (49). Almost two thirds of the 300 midwives wanted further training about immunisation. Those who had adequate knowledge and training were more likely to recommend vaccination. A European study also highlighted deficiencies in midwifery education around vaccination which reinforced the perception that vaccination was the doctor's professional responsibility and not the role of the midwife (50). Box 1 provides an example of an approach used in Australia to provide midwives with information and support in relation to COVID-19 vaccination in pregnancy.

BOX 1 **One approach to supporting midwives to promote COVID-19 vaccination in pregnancy.**

The Australian College of Midwives (ACM) is a national not-for-profit membership organisation and the peak professional body for midwives in Australia (51). As COVID-19 vaccination became a national recommendation for all pregnant women, a dearth of information, specific to midwives in their unique role as primary care providers to over 300,000 births per year (52), was evident. Furthermore, the CovMat-Vax national study identified a strong desire from five stakeholder-cohorts (women, their partners, midwives, doctors and midwifery students) for more information on COVID-19 vaccination (53). In an immediate response, ACM coordinated a live panel to disseminate evidence and provide education and tools to midwives to equip them with knowledge and skills to have crucial COVID-19 vaccination conversations with pregnant women. Presented by midwifery leaders, the event drew over 1,000 registrations for the live 1-hour event. Through the questions that ensued it became apparent that midwives felt very underprepared to have crucial conversations with women regarding COVID-19 vaccinations in pregnancy due to a lack of understanding of the data and evidence and an absence of training being offered within their own health networks.

This need for resources led ACM to design three resources to support midwives in their discussion, education, and recommendation of COVID-19 vaccination for pregnant women. The resources form part of an evidence-based toolkit for midwives to engage with women in shared decision making. ACM purpose-built an online repository consisting of up-to-date real-world evidence and an eLearning course that delivers analysis of the latest evidence and provides midwives with the knowledge and tools to counsel the women in their care about the options available to them.

The eLearning course has been tailor made for Australian midwives and is freely available to all midwives (54). The course includes resources to address vaccine hesitancy in midwives including a reminder of their Code of Conduct, wellbeing resources for self-care, guidance for midwives to support women to be vaccinated, downloadable resources such as talking sheets, infographics and resources to share with women and families, infographics and animations demonstrating how the vaccination works, resources for privately practising midwives, links to Commonwealth resources and professional clinical guidance.

**Figure 1:**
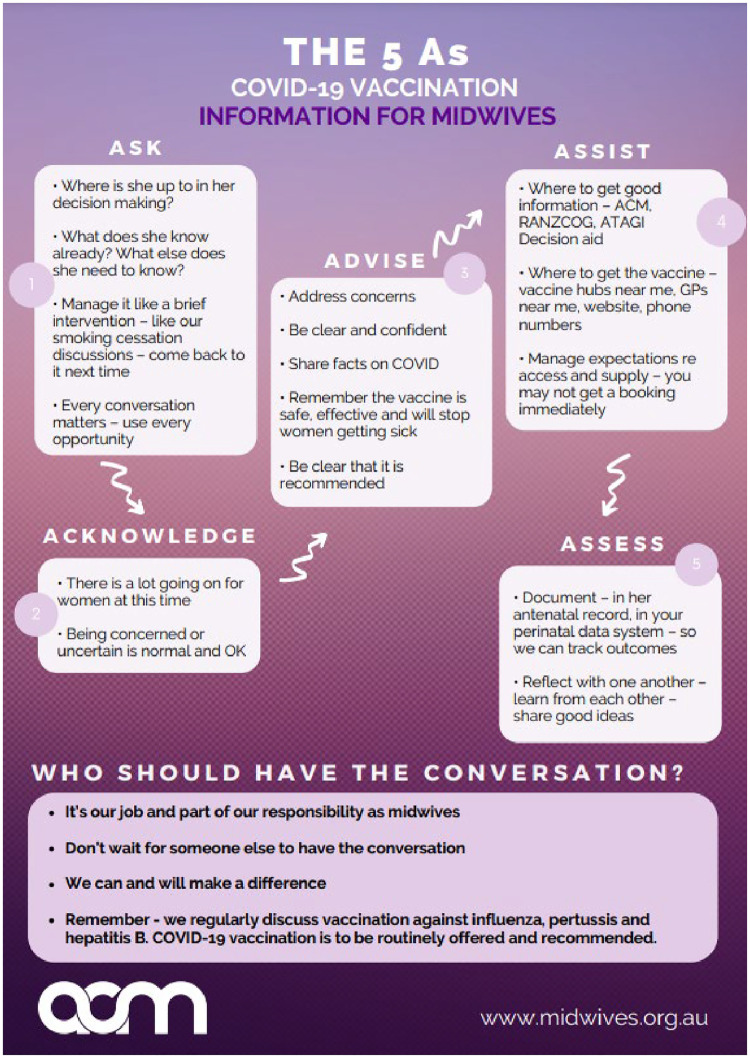
COVID-19 vaccination information for midwives.

Figure 1 provides an outline of the COVID-19 vaccination information for midwives.

Including maternal immunisation in usual antenatal care is a useful strategy that makes the ole of the midwife critical. For example, a study in 95 low and middle income countries showed that the majority utilised antenatal care services to deliver tetanus vaccines (55). Vaccination was provided by midwives or registered, enrolled or auxiliary nurses as part of usual antenatal care. Countries that had good integration between the Expanded Programme on Immunization (EPI) and the usual antenatal care programs were more effective at delivering these vaccination services. The study did, however, show that in many facilities, there was only one health worker responsible for both vaccinations and antenatal care which puts pressure on these individuals especially if additional vaccinations are recommended (55). Ensuring that midwives are part of integrated teams delivering antenatal care and maternal vaccination is important especially in countries where midwives deliver a considerable proportion of antenatal care.

Equipping midwives and other antenatal care providers to discuss the different vaccines with pregnant women is important. Ensuring adequate time to educate midwives in readiness is likely to have benefits. For example, a study in Australia showed that taking time to consult with midwives in advance of a new pertussis vaccination program ensured that the right information was given to women and the program could be successfully implemented (56). A similar program in the United Kingdom was implemented where a group of midwives received additional training and attended influenza and pertussis vaccination clinics throughout the influenza season as well as attending the labour wards and maternity inpatient wards to provide vaccinations to those women who were inpatients in hospital. This has been highly successful in ensuring high uptakes of pertussis vaccination in pregnancy (57).

Education of midwives is necessary to ensure that they are able to make clear recommendations to women in relation to vaccination. Our work with maternity care providers in relation to COVID-19 vaccination in pregnancy showed that doctors and midwifery students were significantly more likely to recommend the vaccine to pregnant women in their care than midwives (53). In our study, more than half of the midwives (53%) had concerns about the COVID-19 vaccine for the women in their care compared with 35% of doctors and 46% of midwifery students. In relation to promoting childhood vaccinations, a systematic review showed that the majority of midwives supported vaccination, although there was a spectrum of beliefs and concerns (58). This again highlights the need for education and training on the need for vaccination, safety of relevant vaccines for the pregnant woman and her baby, and the way to communicate vaccine need and safety of the vaccine for the expected baby with parents. Professional development for midwives should include opportunities for open and honest bi-directional communication where questions can be raised and addressed with scientific evidence to support vaccination conversations in the context of primary maternity care.

The model of antenatal care service may also have an impact on the quality of the discussion between midwife and women and on the uptake. For example, a study of COVID-19 vaccination showed that uptake rates were 12% higher among women receiving continuity of care from a known midwife, than for those receiving standard public maternity care with a range of caregivers not known to the woman (59). Midwifery continuity of care is underpinned by factors known to support vaccination uptake such as formation of a trusting partnership with the woman, and repeated visits with the same person, allowing conversations to continue over the continuum of pregnancy. The trust between women and their midwives in this model, enable health promotion over a period of several visits where conversations are based in an understanding of the woman's identified priorities and evidence-informed decision making. Insights gained from this study (49) warrant further implementation research on the impact of midwifery continuity of care with sub-groups that typically have lower vaccination rates such as women who experience social disadvantage, poverty and unstable housing (26).

The role of the midwives in providing advice regarding vaccination as well as being able to vaccinate at point and time of care have been identified as important strategies to increase vaccination uptake (45). Developing standing orders for midwife or nurse-administered vaccination and making it easy for the vaccination to occur have been shown to be successful in increasing uptake (12). Midwives should be able to provide clients with information and provide vaccination without additional barriers of separate appointments, additional referrals and prescriptions. The keystone role that midwives play in the success of global perinatal vaccination programs, warrants the removal of systemic barriers to supported and qualified midwife prescribing and administration of relevant vaccines at point of care.

## Conclusion

The COVID-19 vaccination programs have shone a light on vaccination in pregnancy. Health care providers are key to the uptake of vaccination in pregnancy. In many countries, midwives play a unique role in the provision of care to women and families and so are a critical resource in the promotion of vaccination in pregnancy.

Midwives in all countries are uniquely placed to assist pregnant women in immunisation decision (60). They often lack the knowledge and confidence to promote vaccination. Midwives, like all health providers, need specific support and information regarding vaccination in pregnancy. Targeted education, supportive mentoring and supervision and opportunities to lead innovative ways of ensuring vaccine access is logistically easy and possible are all needed. This paper has highlighted a number of strategies to enable midwives to promote vaccination in pregnancy. These strategies were always important, especially for influenza, pertussis and tetanus but now are even more essential in the COVID-19 pandemic.

## Data Availability

The original contributions presented in the study are included in the article/Supplementary Material, further inquiries can be directed to the corresponding author/s.
